# Cervical human papillomavirus infection among female sex workers in southern Vietnam

**DOI:** 10.1186/1750-9378-3-7

**Published:** 2008-04-23

**Authors:** Brenda Y Hernandez, Thuong Vu Nguyen

**Affiliations:** 1 Cancer Research Center of Hawaii, University of Hawaii, Honolulu, Hawaii, USA; 2 Pasteur Institute, Ho Chi Minh City, Vietnam

## Abstract

**Background:**

Cervical cancer is the most frequently diagnosed malignancy among women in southern Vietnam where its incidence is one of the highest observed worldwide.

**Results:**

Cervical HPV DNA infection was measured in a cross-sectional sample of 282 female sex workers (FSW) in Soc Trang province in southern Vietnam. HPV DNA was detected in 85% of FSW and prevalence did not vary by age. Thirty-five HPV genotypes were detected; HPV 52 was the most common type. Half of HPV-positive women were infected with oncogenic types and 37% were infected with multiple genotypes. The prevalence of oncogenic HPV infection was lower among FSW with more formal education (adj. prevalence ratio = 0.63, 95% CI 0.42–0.93), those servicing 25 or more clients per month (adj. PR = 0.66 95% CI 0.48–0.92), and those engaging in withdrawal prior to ejaculation (adj. PR = 0.68, 95% CI 0.53–0.87). Oncogenic HPV prevalence was higher among FSW with regular male partners who had other female partners (adj. PR = 1.75, 95% CI 1.34–2.28) and FSW who were HIV+ (adj. PR = 1.42, 95% CI 1.08–1.88).

**Conclusion:**

Our results demonstrate that although cervical HPV infection is extremely common among FSW in southern Vietnam, prevalence varies by education level, sexual activity, habits of regular partners, and HIV status.

## Background

Cervical cancer is the most frequently diagnosed malignancy among women in southern Vietnam where its incidence is one of the highest observed worldwide [1,2]. There is considerable variation in the incidence of cervical cancer within Vietnam with rates in southern regions (age-standardized rate 26 per 100,000) four times higher than that observed in northern provinces (6.1 per 100,000) [1]. These differences correspond to regional differences in the prevalence of HPV, the principal cause of cervical cancer [3], which is substantially higher in southern compared to northern Vietnam [4]. This cross-sectional study examined the genotype-specific prevalence of HPV infection among female sex workers (FSW) in southern Vietnam–a group presumably at particularly high risk for HPV and other sexually transmitted infections.

## Results

Between April and August of 2003, 406 women were recruited. Of these women, interviews, physical exams, and cervical specimen collection were completed for 395. Following chlamydia and gonorrhea testing on 395 cervical DNA specimens, 300 specimens remained and were sent to the HPV testing lab. DNA was sufficient for 282 of the 300 specimens. Sexually transmitted infections other than HPV were evaluated in a recent report. (Nguyen TV, Khuu NV, Le TT, Nguyen AP, Cao V, Tham DC, Detels R. *Submitted*.)

**Figure 1:**
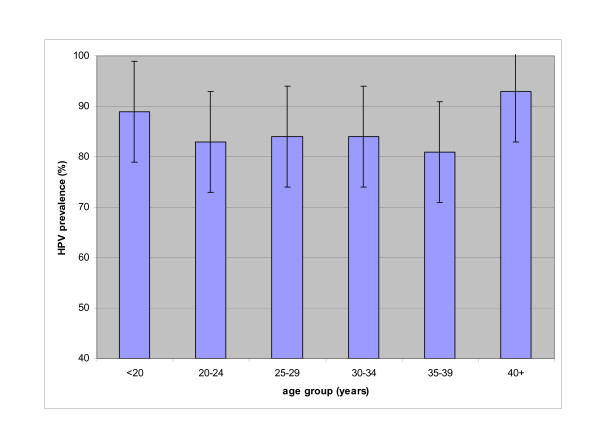
Prevalence of cervical HPV in sex workers by age, Soctrang province, Vietnam.

HPV DNA was detected in 85% (239/282) of women. HPV prevalence varied little by age and exceeded 80% for all age groups (Figure 1). Thirty-five different HPV genotypes were identified. Half (119/239) of HPV-positive women were infected with oncogenic types and 37% (88/239) were infected with multiple genotypes. Oncogenic HPV 52 was the most common type observing comprising 11.4% of all genotypes detected (Table 1). HPV 16, 58, 62, 51, and 18 were also among the most common types. Twenty-two percent of HPV-positive specimens were not positive for any of the 37 types covered in the assay.

**Table 1:** Genotypes detected in cervical HPV-positive^1 ^sex workers, Soc Trang province, Vietnam.

HPV genotype	Frequency	%
52^2^	50	11.4
16^2^	29	6.6
58^2^	19	4.3
62	19	4.3
51^2^	18	4.1
18^2^	17	3.9
59^2^	13	3.0
11	12	2.7
39^2^	11	2.5
84	11	2.5
56^2^	10	2.3
70	10	2.3
66^2^	9	2.1
68^2^	9	2.1
54	8	1.8
55	8	1.8
61	8	1.8
71	8	1.8
6	7	1.6
33^2^	6	1.4
53^2^	6	1.4
72	6	1.4
81	6	1.4
26^2^	5	1.1
73^2^	5	1.1
CP6108	5	1.1
45^2^	4	0.9
83	4	0.9
40	3	0.7
42	3	0.7
67	3	0.7
31^2^	2	0.5
35^2^	2	0.5
82^2^	2	0.5
64	1	0.2
Untyped	99	22.6

Total	438	100

^1^239 HPV+ sex workers; 88 HPV+ for 2 or more genotypes

^2^Oncogenic/probable oncogenic genotypes

The mean age of FSW was 25 years (range 16–47). The majority of FSW was of Kinh ethnicity and over half lived within the local province. Overall, 52% of women were single, 10% were married, 14% were cohabitating, 23% were divorced/separated, and 1% were widowed. Fifty-four percent of women had a current relationship with a regular male partner outside of their commercial sex work. Sixty-three percent of women had no more than a primary school education. Sixty-three percent of women had been a sex worker for less than 3 years. FSW were distinguished by the venues for selling sex. Forty-eight percent of women engaged in sex work from "direct" venues including the streets, parks, bus stops, boats, ferry piers, brothels, small cafés, and guesthouses; 52% worked out of "indirect" venues, that is, hotels, restaurants, massage parlors, karaoke lounges, bars, and barbershops. The mean number of clients seen per month was 18 (range 1–81). All women engaged in vaginal intercourse. Condom use with regular and irregular clients was reported by 84% and 85% of FSW, respectively. Prevalence of sexually transmitted infections other than HPV ranged from 2% for HIV to 43% for chlamydia.

When adjusted for age alone, none of the variables were significant for any HPV infection, oncogenic HPV, or multiple HPV infection. Variables were then evaluated in models containing all 26 variables (Table 2). Variables significant in one or more full models included the following: education level (no formal schooling, primary school, secondary/high school), number of clients serviced per month (<10, 10–25, >25), engaging in withdrawal prior to ejaculation (ever/never), engaging in anal intercourse (ever/never), use of intrauterine device (IUD) (ever/never), age of regular male partner (<30, ≥30), relationship with a regular partner with other female partners (yes/no), and HIV infection (positive/negative).

**Table 2:** Cervical HPV infection and characteristics of sex workers, Soc Trang province, Vietnam

	No. (%)	Any HPV Adj. PR^1 ^(95% CI)	Oncogenic HPV Adj. PR^1 ^(95% CI)	Multiple HPV Adj. PR^1 ^(95% CI)
Age (yrs.)				
<20	45 (16%)	1.00	1.00	1.00
20–29	175 (62%)	0.83 (0.62–1.10)	0.72 (0.48–1.09)	0.72 (0.41–1.25)
30+	62 (22%)	0.78 (0.53–1.16)	0.79 (0.33–1.87)	0.88 (0.37–2.10)
		p trend = 0.35	p trend = 0.12	p trend = 0.07
				
Ethnicity				
Kinh	230 (82%)	1.00	1.00	1.00
Other	52 (18%)	1.14 (0.92–1.41)	1.10 (0.73–1.64)	1.02 (0.60–1.73)
				
Marital Status				
Not Single	136 (48%)	1.00	1.00	1.00
Single	146 (52%)	1.02 (0.83–1.27)	1.29 (0.83–1.98)	1.68 (0.83–6.79)
				
Education				
None	43 (15%)	1.00	1.00	1.00
Primary School	136 (48%)	0.94 (0.73–1.22)	0.78 (0.48–1.25)	0.63 (0.37–1.08)
Secondary-high school	103 (37%)	0.80 (0.59–1.10)	0.57 (0.32–1.04)	0.43 (0.23–0.79)
				
Age of regular partner (yrs.)^2^				
<30	72 (47%)	1.00	1.00	1.00
30+	81 (53%)	1.39 (1.09–1.76)	1.60 (1.13–2.27)	1.78 (1.14–2.78)
				
Regular partner has other female partners^2,3^				
No	54 (36%)	1.00	1.00	1.00
Yes	97 (64%)	1.27 (1.04–1.54)	2.01 (1.34–3.00)	2.17 (1.30–3.62)
				
Age at first sex^4^				
ὄ 15 yrs	11 (4%)	1.00	1.00	1.00
>15 yrs	270 (96%)	0.84 (0.58–1.20)	1.09 (0.54–2.19)	0.77 (0.35–1.69)
				
Duration of commercial sex work				
<1 year	54 (19%)	1.00	1.00	1.00
1 – 3 years	125 (44%)	1.11 (0.79–1.57)	0.88 (0.61–1.27)	0.97 (0.65–1.45)
> 3 years	103 (37%)	1.16 (0.78–1.72)	0.96 (0.62–1.49)	1.20 (0.76–1.88)
		p trend = 0.11	p trend = 0.02	p trend = 0.03
				
Venue for sex work^5^				
Indirect venues	148 (52%)	1.00	1.00	1.00
Direct venues	134 (48%)	1.03 (0.87–1.22)	1.11 (0.77–1.59)	0.99 (0.68–1.44)
				
No. of clients per month (average)				
<10	97 (34%)	1.00	1.00	1.00
10–25	85 (30%)	1.00 (0.88–1.15)	1.08 (0.79–1.46)	1.27 (0.75–2.13)
>25	100 (36%)	0.64 (0.49–0.83)	0.43 (0.27–0.70)	0.52 (0.29–0.94)
		p trend = 0.16	p trend = 0.85	p trend = 0.69
				
Oral-penile contact				
Never	239 (85%)	1.00	1.00	1.00
Ever	43 (15%)	1.29 (0.94–1.78)	1.45 (0.74–2.82)	1.51 (0.70–6.55)
				
Anal-penile contact				
Never	272 (96%)	1.00	1.00	1.00
Ever	10 (4%)	0.72 (0.40–1.34)	0.36 (0.14–0.92)	0.26 (0.10–0.69)
				
Condom use with regular clients^6^				
Not always	37 (16%)	1.00	1.00	1.00
Always	190 (84%)	1.13 (0.88–1.44)	1.58 (0.98–2.55)	1.68 (0.84–3.36)
				
Condom use with irregular clients^7^				
Not always	29 (13%	1.00	1.00	1.00
Always	189 (87%)	0.96 (0.70–1.30)	0.95 (0.69–1.30)	0.94 (0.60–1.49)
				
Oral contraceptives^4^				
Never	261 (93%)	1.00	1.00	1.00
Ever	20 (7%)	1.06 (0.81–1.40)	1.32 (0.84–2.07)	1.41 (0.75–2.64)
				
Intrauterine device^4^				
Never	218 (78%)	1.00	1.00	1.00
Ever	63 (22%)	0.89 (0.69–1.13)	0.63 (0.33–1.20	0.52 (0.28–0.99)
				
Withdrawal^4^				
Never	198 (70%)	1.00	1.00	1.00
Ever	83 (30%)	0.80 (0.67–0.96)	0.62 (0.42–0.89	0.58 (0.30–1.12)
				
Illegal drugs^4^				
Never	255 (91%)	1.00	1.00	1.00
Ever	26 (9%)	0.76 (0.54–1.06)	0.61 (0.33–1.13)	1.42 (0.80–2.52)
				
Pregnant^4^				
Never	144 (51%)	1.00	1.00	1.00
Ever	138 (49%)	1.00 (0.81–1.24	0.95 (0.61–1.46)	1.06 (0.59–1.92)
				
Current STI status^4^				
Trichomonas				
Negative	258 (92%)	1.00	1.00	1.00
Positive	23 (8%)	1.04 (0.78–1.39)	1.30 (0.84–2.02)	1.51 (0.76–3.00)
				
Bacterial vaginosis				
Negative	200 (71%)	1.00	1.00	1.00
Positive	81 (29%)	1.10 (0.93–1.31)	1.07 (0.75–1.52)	1.30 (0.58–2.00)
				
Yeast				
Negative	253 (90%)	1.00	1.00	1.00
Positive	28 (10%)	1.20 (0.97–1.49)	0.80 (0.51–1.25)	0.81 (0.39–1.69)
				
Syphilis				
Negative	270 (96%)	1.00	1.00	1.00
Positive	11 (4%)	1.34 (0.84–2.16)	1.38 (0.65–2.93)	1.05 (0.42–2.67)
				
HIV				
Negative	276 (98%)	1.00	1.00	1.00
Positive	5 (2%)	1.57 (0.98–2.51)	3.01 (1.01–9.00)	2.87 (0.69–12.00)
				
Gonorrhea				
Negative	253 (90%)	1.00	1.00	1.00
Positive	28 (10%)	1.06 (0.91–1.24)	0.92 (0.89–1.45)	0.98 (0.68–2.86)
				
Chlamydia				
Negative	161 (57%)	1.00	1.00	1.00
Positive	120 (43%)	0.99 (0.76–1.28)	0.95 (0.66–1.37)	0.98 (0.64–1.50)

^1^Prevalence ratio, adjusted for all variables

^2^153 FSW had a regular male partner

^3 ^Responses missing from 2 subjects

^4 ^Response missing from 1 subject

^5 ^Direct venues include streets, parks, bus stops, boats, ferry piers, brothels, small cafés, and guesthouses; indirect venues include hotels, restaurants, massage parlors, karaoke lounges, bars, and barbershops.

^6 ^227 FSW serviced regular clients

^7 ^218 FSW serviced irregular clients

Final models were developed consisting of these 8 variables in addition to age (continuous) (Table 3). FSW with a secondary or high school education were less likely to be positive for oncogenic or multiple HPV types than women with a primary school education or no formal schooling. FSW servicing 25 or more clients per month were less likely to have any or oncogenic HPV infection compared to those with fewer clients. The prevalence of any, oncogenic, and multiple HPV infection was significantly lower among FSW engaging in withdrawal prior to ejaculation. FSW using IUDs were less likely to be infected with multiple HPV types. The prevalence of HPV (any, oncogenic, and multiple infection) was significantly greater among HIV+ women. Apart from their commercial sex work, FSW with regular male partners who had other female partners were more likely to have all 3 HPV outcomes while those with male partners ages 30 years and older were more likely to have any infection.

**Table 3:** Factors associated with cervical HPV infection in sex workers, Soc Trang province, Vietnam

	Any HPV Adj. PR^1 ^(95% CI)	Oncogenic HPV Adj. PR^1 ^(95% CI)	Multiple HPV Adj. PR^1 ^(95% CI)
Education			
None	1.00	1.00	1.00
Primary School	0.92 (0.73–1.15)	0.80 (0.58–1.11)	0.82 (0.56–1.21)
Secondary-high school	0.80 (0.61–1.04)	0.63 (0.42–0.93)	0.61 (0.38–0.99)
			
No. of clients per month (average)			
<10	1.00	1.00	1.00
10–25	1.03 (0.92–1.16)	1.06 (0.87–1.30)	1.14 (0.83–1.57)
>25	0.78 (0.65–0.94)	0.66 (0.48–0.92)	0.70 (0.47–1.06)
	p trend = 0.93	p trend = 0.43	p trend = 0.49

Regular partner has other female partners^2,3^			
No	1.00	1.00	1.00
Yes	1.22 (1.03–1.44)	1.75 (1.34–2.28)	1.91 (1.41–2.60)
			
Intrauterine device^4^			
Never	1.00	1.00	1.00
Ever	0.85 (0.68–1.06)	0.58 (0.31–1.08)	0.53 (0.29–0.99)
			
Withdrawal^4^			
Never	1.00	1.00	1.00
Ever	0.84 (0.73–0.97)	0.68 (0.53–0.87)	0.60 (0.43–0.83)
			
HIV^4^			
Negative	1.00	1.00	1.00
Positive	1.20 (1.01–1.42)	1.42 (1.08–1.88)	1.45 (1.01–2.10)
			
Age of regular partner (yrs.)^2^			
<30	1.00	1.00	1.00
30+	1.21 (1.03–1.44)	1.16 (0.92–1.46)	1.22 (0.86–1.73)

^1^Adjusted for all variables listed in addition to age, condom use with regular clients, gonorrhea, sex work venue

^2^153 FSW had a regular male partner

^3 ^Responses missing from 2 subjects

^4 ^Response missing from 1 subject

## Discussion

Our results demonstrate that cervical HPV infection is extremely common among southern Vietnamese sex workers with an overall prevalence nearly twice that of chlamydia. HPV prevalence in the present study was much higher than that observed in a population-based prevalence study of HPV in Vietnam [4]. In Ho Chi Minh City, the major city of southern Vietnam, 10.9% of women were positive for cervical HPV DNA compared to 2.0% of women in Hanoi, the major city of northern Vietnam [4]. The prevalence of HIV among sex workers in Vietnam shown similar geographic disparities with rates in the southern border higher than that in the north and central regions [5].

The basis for these differences in the prevalence of HPV and other sexually transmitted infections by geographic region is not completely understood. It may reflect differences in sexual practices including the pervasiveness of prostitution by region. Soc Trang province, where nearly 1/3 of the population is Khmer, is proximate to Kien Giang, An Giang and Dong Thap provinces which border Cambodia. Sex workers commonly traverse between the 2 countries through these areas. It is possible that these highly transient sex workers are at particularly high risk for HPV. The substantial proportions of multiple infections and untyped specimens are consistent with a high-risk population with exposure to many different partners.

Oncogenic HPV 52 was more common than HPV 16 in this population of sex workers. HPV 52 is one of the most common types found among women in southeast Asia as well as eastern Asia including women with normal cervical cytology as well as those with SIL and carcinoma [6]. HPV 16 is estimated to be responsible for the majority of invasive cervical cancers. It is not known whether the proportion of cervical cancers attributed to HPV 16 in southern Vietnam, which has among the highest rates of cervical cancer in the world [1,2], is different from other parts of the world.

The lack of variation of HPV prevalence with age among these sex workers is contrary to what has been observed in North American, European, and some Asian populations. The flat age curve in this population of sex workers is similar to that observed in high-risk populations in India and Nigeria characterized by high cervical cancer incidence and mortality [7].

The observed association of education level with oncogenic and multiple-type HPV infection is consistent with studies in other countries demonstrating an inverse association of socioeconomic status and HPV [8,9]. Education and other indicators of socioeconomic status may reflect differences in sexual practices, partner characteristics, and other factors that influence exposure to the virus.

Interestingly, HPV infection (any and oncogenic types) were less prevalent among FSW servicing the most clients. Risk of cervical HPV infection has been consistently observed to increase with number of recent partners [10] and partners over the lifetime [11]. In contrast to the general female population, however, sex workers are likely to have consistent and repeated exposure to HPV with each new partner. It has been suggested that persistent, repetitive exposure to HPV antigen increases the development and persistence of local immune responses [12]. Accordingly, those sex workers with the largest number of clients may be most protected from acquisition of new HPV infections. Consistent with this theory, compared to more experienced sex workers in Mexico City, an increased risk of HPV infection was observed among women involved in prostitution for less than 1 year [13].

The lower prevalence of HPV among FSW engaging in withdrawal during intercourse is difficult to explain. Semen is not a major site of HPV infection in males compared to the external genitals [14]. Furthermore, use of withdrawal is likely correlated with lack of use of condoms and other contraceptive methods. We also observed a lower prevalence of multiple HPV infection among IUD users. Previous studies have demonstrated a protective effect of IUD use with abnormal cervical cytology and cervical adenocarcinoma [15,16].

The prevalence of oncogenic and multiple HPV was higher among the limited number of HIV-positive women in this population. This is consistent with studies of cervical HPV and HIV in other populations [17,18] and may reflect the increased susceptibility to viral acquisition and/or to viral persistence with HIV-induced immune suppression.

Our results demonstrate that even among a high-risk population such as commercial sex workers, the behavior of regular partners, including spouses and cohabitating partners, plays an important role in HPV acquisition. Sex workers with regular male partners who engaged in sexual relationships with other females were at increased risk of any, oncogenic, and multiple HPV infection. While other studies have demonstrated that the important role of men who are serviced by prostitutes in transmission of HPV to their wives [11,19], the present study demonstrates that even for high-risk females, the behavior of their regular male partners are key to the acquisition of the virus. This may indicate that HPV transmission is most efficient with repeated exposure to the same source relative to one-time or periodic exposures.

Interestingly, the relationship of partner's age and HPV prevalence contrasts with other sexually transmitted infections. A previous study of sex workers in Vietnam found that age of partners was inversely associated with risk of gonorrhea and chlamydia [20]. It is possible that HPV is a more persistent infection in older men such that older partners are more likely to transmit infections.

Observations of the role of condoms in HPV infection are inconsistent and conflicting [11,21,22]. The large majority of sex workers reported 100% use of condom with regular clients. However, information on condom use with regular partners was not collected. It is possible that condoms were less likely to be used with regular partners thereby increasing the risk of acquiring sexually transmitted infections from those partners who had other sexual relationships.

Given the transient nature of most HPV infections, this cross-sectional study offers a limited perspective of the natural history of HPV in this high-risk population. The minority of sex workers who were negative for HPV in the present study were likely infected in the past and subsequently cleared these HPV infections. The HPV-positive sex workers observed in this study may disproportionately include those with persistent infections. The ability to distinguish factors associated with any, oncogenic, and multiple infection was limited as many women were positive for multiple HPV types including concurrent oncogenic and non-oncogenic types.

## Conclusion

The dynamics of HPV transmission are not well understood. Our study demonstrates that sex workers may be important reservoirs of HPV infection within southern Vietnam where the incidence of cervical cancer is one of the highest observed worldwide. Effective measures to reduce the incidence of invasive cervical cancer in the population will ideally incorporate both primary and secondary prevention efforts, including HPV vaccination and cervical screening.

## Methods

### Study Recruitment

The study was approved by the Pasteur Institute, Ho Chi Minh City and the Standing Bureau for HIV/AIDS in Soc Trang province (currently known as the Soc Trang Center for HIV/AIDS Control and Prevention).

Initially, a mapping process to identify local sites where FSW could be found and the numbers of FSW in each hot spot was conducted by meeting with various local provincial and district sector officials, including health workers, police, the women's union, the Department of Labor, Invalid and Social Affairs, and peer educators.

Using the information from mapping, 406 FSW in Soc Trang province were identified and invited to participate in a cross-sectional study between May and August 2003. Because no lists of FSW were available, it was impossible to do systematic random sampling to recruit subjects in each hot spot. After obtaining agreement from the owners of establishments and FSW, subjects were recruited by convenience sampling. The number of refusals (FSW who avoided meeting with the interviewers) was not known. Verbal informed consent was obtained from all study subjects.

### Specimen collection

Blood specimens were obtained for serological measurement of syphilis (rapid plasma regain; Bio-Rad) and HIV (SFD HIV 1/2 PA, Bio-Rad). Positive sera were first stored at -20°C, then batched and sent to the Pasteur Institute in Ho Chi Minh City every two weeks for confirmation with two ELISA tests for HIV (Murex HIV 1.2.0, Abbott; Genscreen HIV 1/2 V.2, Bio-Rad) and the *Treponema pallidum *hemagglutination assay (Bio-Rad) for syphilis.

Vaginal fluid specimens were obtained for microscopic and clinical identification of trichomoniasis and candidiasis. Bacterial vaginosis was defined by Amsel criteria. Cervical secretions were collected and temporarily stored at -20 degrees C prior to transport to the Pasteur Institute in Ho Chi Minh City where they were stored at -70 degrees C. DNA was extracted from cervical specimens and used for PCR-based testing of chlamydia, gonorrhea, and HPV.

### Interview

Study subjects were interviewed by trained interviewers using a structured questionnaire. Social and demographic information and behavioral and sexual characteristics were queried.

### HPV DNA testing, genotyping, and variant analysis

Frozen cervical DNA was sent to the Cancer Research Center of Hawaii in Hawaii, U.S.A. for HPV DNA testing and genotyping. The PCR reaction utilized PGMY09/PGMY11 primers [23] to amplify a 450 base pair region of the L1 HPV genome (nt 6582–7033).

HPV-positive specimens were subsequently genotyped using a reverse line blot detection method [24] (Roche Molecular Systems) for 37 different HPV types including oncogenic/probable oncogenic types (HPV 16, 18, 26, 31, 33, 35, 39, 45, 51, 52, 53, 56, 58, 59, 66, 68, 73, 82, IS39), non-oncogenic types (HPV 6, 11, 40, 42, 54, 61, 70, 72, 81, CP6108), and types with undetermined risk status (HPV 55, 62, 64, 67, 69, 71, 83, 84) [25,26]. HPV-positive specimens that were subsequently found to be negative in the genotyping assay were considered to be untyped HPV-positive specimens. The HPV testing and genotyping procedure has been detailed previously [27].

All specimens were also tested using GH20 and PC04 primers to amplify a 268 base pair region of the human beta-globin gene as an internal control for sample sufficiency. Specimens testing negative for beta-globin were considered to be insufficient and were excluded from analyses.

### Statistical analysis

SAS software version 9.1 (SAS Institute, Inc.) was used for all data analyses. The relationship of variables with HPV infection was evaluated using prevalence ratios (PRs). PRs and 95% confidence intervals (CI) were estimated using modified Poisson regression with robust error variance, which produces estimates with minimal bias and is particularly suited for outcomes of high prevalence [28]. Multivariate models were generated for 3 outcomes: 1) any HPV, 2) oncogenic HPV (including probable oncogenic types), and 3) multiple HPV types (positivity for more than 1 HPV type).

## Competing interests

The authors declare that they have no competing interests.

## Authors' contributions

All authors read and approved the final manuscript. Dr. Nguyen was responsible for the overall design and conduct of the study in Vietnam including staff training, study recruitment, data collection, laboratory testing, and data management. Dr. Hernandez was responsible for HPV testing and genotyping. Dr. Nguyen and Hernandez were both responsible for the statistical analysis of data and the authorship of this manuscript.
